# Effects of extreme air pollution and El Niño Southern Oscillation on insufficient sleep: a cross-sectional study

**DOI:** 10.1186/s12889-025-23316-9

**Published:** 2025-06-04

**Authors:** Haris Majeed, Daniyal Zuberi

**Affiliations:** University of Toronto, Toronto, Ontario M5S 1V4 Canada

**Keywords:** Sleep, Environment, Air pollution, El Niño Southern Oscillation

## Abstract

**Background:**

While there are many well understood clinical risk factors on sleep patterns, the associations of environmental factors, specifically air pollution on insufficient sleep remains understudied. This study investigates the association between insufficient sleep and particulate matter 2.5 (PM_2.5_) among adults in the United States. There is also a need to determine whether various El Niño Southern Oscillation (ENSO) phases are effect modifiers in this relationship.

**Method:**

A cross-sectional observational analysis using annual survey data from 3100 United States counties for adult (≥ 18 years) age-adjusted insufficient sleep prevalence from 2017 to 2024. Annual average county-specific PM_2.5_ data was categorized into three categories [low (< 5 µg/m^3^), moderate (5–11 µg/m^3^), extreme (≥ 11 µg/m^3^)]. The annual average ENSO index was used to determine if the year was either El Niño, La Niña, or neutral. Adjusted associations were conducted using Poisson regression and were stratified by various phases of ENSO. Adjusted associations were reported as rate ratio (RR).

**Results:**

From 2017 to 2024, the United States annual insufficient sleep is 34% [range min to max: 23–49%]. With respect to low PM_2.5_; moderate and extreme PM_2.5_ levels were associated with an increased risk of insufficient sleep by 1.03 (95% CI 1.02–1.05, *P* < 0.001) and 1.11 (95% CI 1.09–1.12, *P* < 0.001), respectively. The interaction between PM_2.5_ and ENSO was significant (*P* < 0.001) on insufficient sleep. The magnitude in associations between extreme PM_2.5_ and insufficient sleep differed by various ENSO phases.

**Conclusion:**

Long-term (i.e. annual) effects of air pollution can pose a risk on adult sleep. While El Niño and La Niña phases were found to be a significant effect modifier, yet during the neutral phase the risks for extreme PM_2.5_ were observed to be the strongest on insufficient sleep. Further investigations are needed to recognize the environmental effects on sleep deprivation.

## Significance

What is already known on this topic

What this study adds

How this study might affect research, practice or policy

There are many known risk factors for insufficient sleep among adults. Emerging research has documented new environmental factors which have shown significant effects on insufficient sleep, including air pollution. Extreme levels of atmospheric air pollution have been associated with adult sleep disturbances, especially particulate matter 2.5 (PM_2.5_). But the majority of studies have documented short-term effects of PM_2.5_ (1-hour or daily), ignoring long-term risks of PM_2.5_ on sleep quality. Furthermore, air pollution episodes were strongly associated with climate variability, such as the El Niño Southern Oscillation (ENSO), which contribute to risks on health outcomes. Hence this study seeks to understand the long-term risk of PM_2.5_ on insufficient sleep in the United States and whether ENSO acts as an effect modifier in the relationship between PM_2.5_ and sleep.

In the past eight years, the prevalence of insufficient sleep among adults in the United States has increased by 4.5%. Moderate and extreme PM_2.5_ levels were significantly associated with a risk of 3% and 11%, respectively on insufficient sleep. Due to the significant interaction present between PM_2.5_ and ENSO, models were stratified by ENSO phases. We found that all phases of ENSO significantly increased the risk of extreme PM_2.5_ on adult insufficient sleep in the United States.

This study carries significant implications for research, practice, and policy, highlighting the necessity of nationwide efforts to reduce PM_2.5_ levels. To mitigate adverse health effects, communities should prioritize safety during periods of deteriorating air quality. Furthermore, the predictability of ENSO up to a year in advance offers the potential to forecast and prepare for shifts in sleep behaviors. At the urban level, thoughtful planning and policy changes could lead to improved sleep outcomes and a reduction in sleep disparities.

## Introduction

Insufficient sleep among adults is a public health epidemic that is often unrecognized. In the United States approximately one-third of adults experience insufficient sleep, which is defined as < 7–8 h of sleep per night. [1] There are numerous reasons for insufficient sleep. For instance, behavioral and technological factors include being sedentary and high levels of cell phone or computer use. Higher sedentary time (> 11 h per day) was associated with higher odds of short sleep. [2] Increased use of technology use before bedtime resulted in a lack of sleep or disturbance in sleep patterns. [3] Other factors can include economic influences. Adults that have suffered from unemployment were found to experience insufficient sleep at a higher rate than employed adults. [4] Others have also documented that an increase in the region’s income inequality was associated with reduction in sleep duration [5, 6]. While these factors have been noted as possible risks for insufficient sleep; environmental and climate factors contributing to sleep deprivation require further investigations.

Previous analyses point to the potential importance of environmental and climate factors. A systematic review (1980–2017) found that increase extreme climate events could have effects on human sleep. [7] While another study reviewed recent literature to find that traffic and pollution can influence adult sleep disorders. [8] Extreme levels of atmospheric air pollution has been associated with adult sleep disturbances, specifically particulate matter 2.5 (PM_2.5_) due to its granular size. [9–14] In particular, one study focused on the short-term effects of PM_2.5_ (1-hour periods) found that increasing PM_2.5_ reduced sleep efficiency. [14] Similarly the long-term effects have revealed that each 1 µg/m^3^ in one-year average PM_2.5_ was associated with a statistically significant increase of 61% with insomnia. [11] Furthermore, research points to the impact of climate variability, the El Niño Southern Oscillation (ENSO), on PM_2.5_ levels in the United States. [15] A global study found that air pollution episodes were strongly associated with climate variability, which were associated with up to 14% increase in annual global PM_2.5_ attributable premature deaths. [16] In general, during La Niña phases (i.e. cooler Pacific Ocean) the United States experiences higher and maximum PM_2.5_ concentrations than during El Niño phases. [15, 17] This is because wind speeds are weak during El Niño phases, which can limit atmospheric dust loading from spreading long distances. [15] However other research has shown that during El Niño phases, the sensitivity of PM_2.5_ across the United States has larger variability. [18] A city level (Taipei, Taiwan) study that found long-term effects, suggesting that that each 1 µg/m^3^ in one-year average PM_2.5_ showed a statistically significant increase of 61% with insomnia. [11] This work invoked the interest to study the link between PM_2.5_, ENSO, and insufficient sleep.

While extreme PM_2.5_ is hypothesized to have effects on insufficient sleep, PM_2.5_ long-term (annual) exposure may pose larger threats to sleep quality in comparison to short-term (i.e. hourly or daily) increases. This study seeks to understand the long-term risk of PM_2.5_ on insufficient adult sleep in the United States over nearly the past decade. Furthermore, we understand whether ENSO acts as an effect modifier in the relationship between PM_2.5_ and sleep. The hypothesis is that phases of ENSO will modify the relationship between PM_2.5_ and sleep, where extreme PM_2.5_ may yield a positive association on insufficient sleep.

## Methods

### Outcome and covariate data

An observational-based study was conducted for the pooled years of 2017–2024. The outcome, ‘insufficient sleep’ is defined by the annual percentage of adults (≥ 18 years of age) who report < 7 h of sleep on average (age-adjusted) using Robert Wood Johnson Foundation data (https://www.countyhealthrankings.org/). Direct age-adjusted was applied according to the year 2000 United States standard population, a method employed by CDC. [19] The Robert Wood Johnson Foundation acquires data from the Behavioral Risk Factor Surveillance System (BRFSS), which conducts annual respondents in the institutionalized population > 18 years of age and have included more than 400,000 annual respondents with landline telephones or cellphones since 2011 through surveys and all measures are based on self-reports. [19, 20] County-specific data was obtained for 3100 counties all throughout the United States, along with other covariate measures including prevalence of adult diabetes (%), smoking (%), physical inactivity (%), uninsured (%), college education attainment (%), median household income (USD), unemployment rate (%), census region, and annual trend. The annual trend is the year of survey that was included as a covariate in our model to account for secular trends in sleep insufficiency.

### Exposure data

County-specific annual average PM_2.5_ concentrations (µg/m^3^) were asteriated from CDC’s National Environmental Public Health Tracking Network (https://ephtracking.cdc.gov/DataExplorer/). We now clarify that the PM_2.5_ data were population-level, county-aggregated to average annual daily density of fine particulate matter in micrograms per cubic meter, based on satellite-derived and ground-monitor-corrected. [21].

While there is no set definition of ‘extreme’ air pollution, rather ≥ 95th percentile of data was chosen. The 5th percentile of annual average PM_2.5_ is ~ 5 µg/m^3^, whereas the 95th percentile of PM_2.5_ is ~ 11 µg/m^3^ for all the United States throughout 2017 to 2024. For this reason, PM_2.5_ concentrations were categorized into three categories; low (< 5 µg/m^3^), moderate (5–11 µg/m^3^), extreme (≥ 11 µg/m^3^).

Throughout the same period, we ascertained data from NOAA for the ENSO index. The ENSO’s two dominant phases are El Niño (warm) and La Niña (cool). The ENSO index reflects deviations in sea surface temperatures over the central equatorial Pacific region (5 N-5 S, 170 W–120 W). [22] For each year, the ENSO index was classified into three categories from cooler to warmer values; ≤-0.5 °C (La Niña), >-0.5 °C to < + 0.5 °C (neutral), ≥+0.5 °C (El Niño).

### Statistical analysis

The objective of this study was to understand associations between extreme PM_2.5_ and insufficient sleep in the United States. Data variables, structures, and covariates are described above. The prevalence of insufficient sleep’s variance and mean was approximately equal. Hence the associations between PM_2.5_, ENSO, and insufficient sleep were conducted using Poisson regression. Adjusted models controlled for the following variables; adult diabetes, smoking, physical inactivity, uninsured, education, household income, unemployment rate, region, and trend (year). Categorical ENSO variable was found to have a significant interaction term in the association between PM_2.5_ and insufficient sleep, therefore ENSO was treated as an effect modifier. To account for location-specific random effects, we included the geographic indicator of county to account for this. The estimated parameters of the coefficient were interpreted as a rate ratio (RR), using two-sided Student’s *t*-test where 95% confidence level (*P* < 0.05) was deemed significant.

## Results

**Fig. 1:**
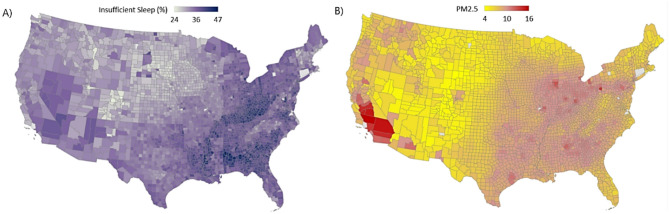
Geospatial variations of average annual (**A**) adult (≥ 18 years) prevalence of insufficient sleep and (**B**) PM_2.5_ concentrations (µg/m^3^) from 2017–2024

From 2017 to 2024 the average adult prevalence of insufficient sleep is 34.3% in the United States (*n* = 3100 counties). Fig. 1 shows the county level variation in sleep and PM_2.5_ across the United States. In 2017, the average adult prevalence of insufficient sleep was 33% and jumped to 34.5% in 2024. This corresponds to a 4.5% increase in eight years with a significant rising trend (*P* < 0.001). Table 1 represents summary statistics for the minimum/maximum, the mean [± standard deviation (SD)], the 5th, 50th (median), and 95th percentiles for each variable used in this study.

**Table 1:** County-level summary statistics of outcome, exposures, and covariates for the United States, 2017–2024

Variable	Minimum	Mean (± SD)	5th Percentile	50th Percentile	95th Percentile	Maximum
Insufficient sleep (%)	22.7	34.3 ± 4.3	27.3	34.3	41.5	49.1
PM_2.5_ (µg/m^3^)	0.9	8.3 ± 1.9	5.1	8.5	11.2	20.9
ENSO index (℃)	-1	-0.05 ± 0.7	-1	0.02	1.1	1.1
Diabetes (%)	1.8	11.3 ± 2.9	8	11	16.6	34.1
Smoking (%)	5.9	18.9 ± 4.1	14.2	18.7	26	43
Physical inactivity (%)	8.1	26.9 ± 5.6	20	26.8	34.2	51.8
College education (%)	0.8	58 ± 11.8	42.8	58	73	100
Household income (US$)	2981	55,479 ± 17,813	38,255	52,019	88,341	243,421
Uninsured (%)	2.5	14.1 ± 6.3	7	13.1	25.6	47
Unemployment rate (%)	0.6	4.8 ± 1.9	2.7	4.4	8.4	24

**Fig. 2:**
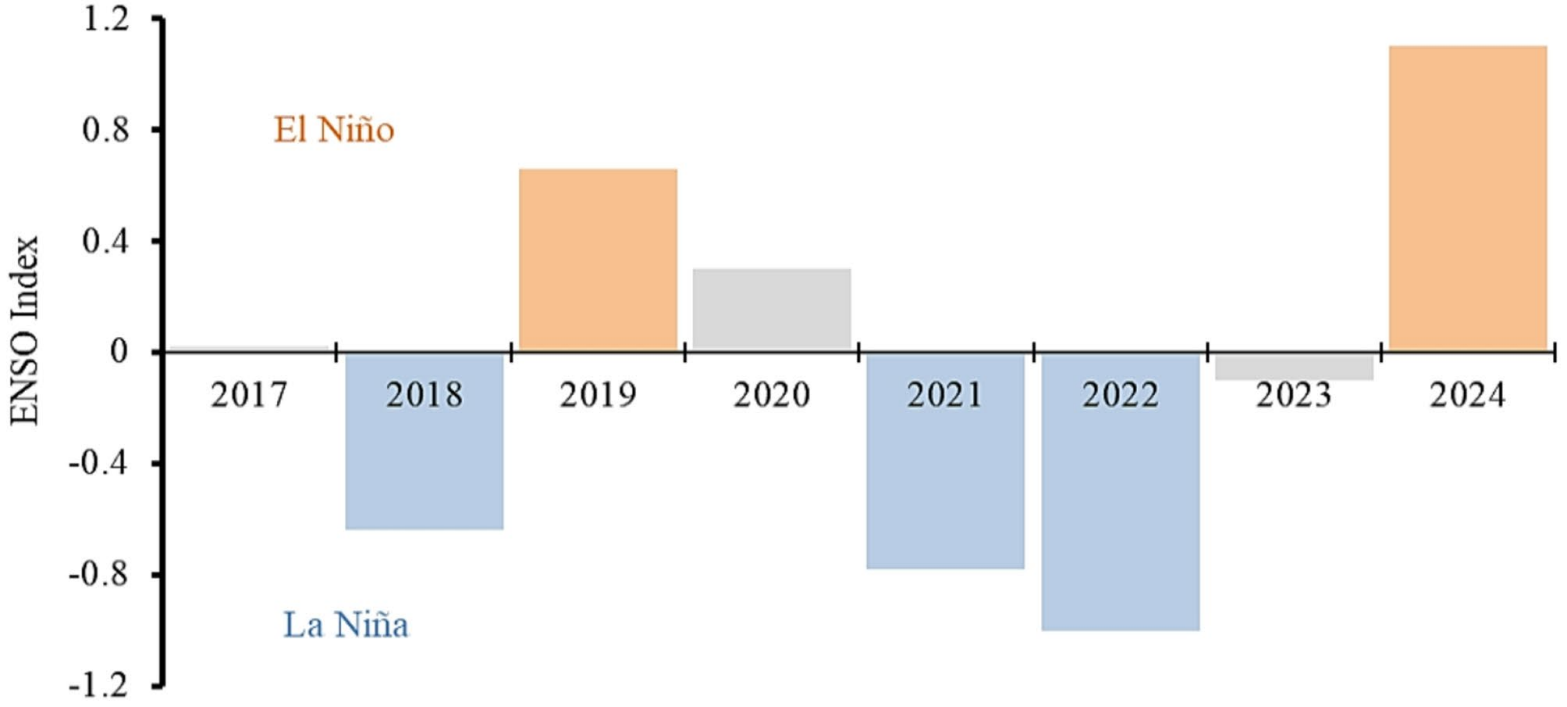
Annual average ENSO index based on central equatorial Pacific sea surface temperature variability from 2017–2024

The annual average ENSO index from 2017 to 2024 is shown in Fig. 2. Years 2018, 2021, and 2022 were identified as La Niña years, whereas 2019 and 2024 as El Niño years. The neutral phases in 2017, 2020, 2023 were used as reference years for our association models.

Associations between insufficient sleep and PM_2.5_ levels were found to be significant. In particular, moderate levels of PM_2.5_ (in comparison to low) were associated with a risk of 1.03 (95% CI 1.02–1.05, *P* < 0.001) on insufficient adult sleep, whereas extreme PM_2.5_ (in comparison to low) was associated with a risk of 1.11 (95% CI 1.09–1.12, *P* < 0.001), shown in Table 2. Furthermore, for ENSO (independent in the model), the predictor of La Niña phase was significantly associated with an increased risk of insufficient sleep (aRR 1.03, 95% CI 1.02–1.03, *P* < 0.001) in the United States, whereas El Niño was not associated. (Table 2). From this table, ENSO and PM_2.5_ were run as separate models. Since there are two level of PM_2.5_ (moderate and extreme) in the model, as well as two levels of ENSO (El Niño and La Niña) in the model, therefore there would be four interaction coefficients. All four interaction term coefficients were negative and were significant except between moderate PM_2.5_ and El Niño. This suggests that different phases of ENSO can act as an effect modifier in the relationship between insufficient sleep and PM_2.5_.

**Table 2:** Associations between insufficient sleep, PM_2.5_, and ENSO for the united States, 2017–2024

Model Predictors	Level	Adjusted RR*	95% CI (*P*-value)
Air pollution (PM_2.5_)	Low (≤ 5 µg/m^3^)	reference	
	Moderate (5–11 µg/m^3^)	1.03	1.02–1.05 (*P* < 0.001)
	Extreme (≥ 11 µg/m^3^)	1.11	1.09–1.12 (*P* < 0.001)
ENSO index	Neutral (>-0.5 °C to < + 0.5 °C)	reference	
	El Nino (≥ 0.5 °C)	0.99	0.98-1.00 (*P* = 0.12)
	La Nina (≤-0.5 °C)	1.03	1.02–1.03 (*P* < 0.001)

* adjusted for adult diabetes, smoking, physical inactivity, uninsured, education, household income, unemployment rate, region, and trend

Associations between sleep and PM_2.5_ were stratified by years in which El Niño, La Niña, and neutral events occurred (Table 3). The magnitude in risk for moderate and extreme PM_2.5_ on sleep was relatively similar for both phases of El Nino or La Nina years. For instance, during both El Niño and La Niña years, the impact of extreme PM_2.5_ is approximately 9% increased risk on insufficient sleep. Whereas, during neutral years, the risk of extreme PM_2.5_ is significant on insufficient sleep at approximately 14%. This suggests that extreme PM_2.5_ during El Niño and La Niña years can significantly impact insufficient adult sleep in the United States, but during neutral years the effect magnitude that extreme PM_2.5_ can have on sleep patterns could be greater.

**Table 3:** Associations between insufficient sleep and PM_2.5_ during various ENSO phases, 2017–2024

ENSO Phase	PM_2.5_ Level as Predictor	Adjusted RR*	95% CI (*P*-value)
El Nino	Low (≤ 5 µg/m^3^)	reference	
	Moderate (5–11 µg/m^3^)	1.033	1.012–1.053 (*P* = 0.001)
	Extreme (≥ 11 µg/m^3^)	1.095	1.067–1.123 (*P* < 0.001)
Neutral	Low (≤ 5 µg/m^3^)	reference	
	Moderate (5–11 µg/m^3^)	1.052	1.024–1.079 (*P* < 0.001)
	Extreme (≥ 11 µg/m^3^)	1.139	1.106–1.173 (*P* < 0.001)
La Nina	Low (≤ 5 µg/m^3^)	reference	
	Moderate (5–11 µg/m^3^)	1.027	1.007–1.046 (*P* = 0.006)
	Extreme (≥ 11 µg/m^3^)	1.094	1.065–1.123 (*P* < 0.001)

* adjusted for adult diabetes, smoking, physical inactivity, uninsured, education, household income, unemployment rate, region, and trend

## Discussion

This research investigation has found a significant long-term association between extreme PM_2.5_ and insufficient sleep among adults in the United States. We have also found that ENSO is a significant effect modifier in the relationship between moderate and extreme levels of PM_2.5_ on insufficient sleep. To our knowledge there has been no study to understand associations with long-term associations between ENSO and sleep.

We also found a rising trend in insufficient sleep among adults in the United States. This is consistent with a study that notes ascending prevalence of insufficient sleep worldwide from a combination of behavioral, technological and climate/environmental factors. [23, 24].

While no study has found associations with ENSO and sleep, but pertaining to air pollution, our results are consistent with one study that found long-term effects, suggesting that that each 1 µg/m^3^ in one-year average PM_2.5_ showed a statistically significant increase of 61% with insomnia. [11] Sleep efficiency in the highest exposure quintiles of PM_2.5_ was associated with 3.2% (*P* < 0.05) in the United States. [14] Another study has noted that an increase in 1-year mean PM_2.5_ by 3.4 µg/m^3^ was associated with a 4.7% increase in sleep-disordered breathing in Taiwan. [25] We also found that extreme PM_2.5_ significantly impacts insufficient sleep across all ENSO phases, yet its effect is more pronounced during neutral years, possibly due to less favorable conditions for pollutant dispersion and removal via winds or storms.

A definite mechanism to explain the effects of PM_2.5_ on sleep patterns has not yet been identified. High concentrations of PM_2.5_ in the lower atmosphere can disrupt the protective barrier of the airway epithelium and mucosa, potentially leading to inflammation within the airways. [26] This can also induce pyroptosis, compromising the integrity of the air-blood barrier through oxidative stress mechanisms. [27] These plausible mechanism collectively indicate that PM_2.5_ exposure may be a triggering factor in altering sleep stages and increasing arousal during sleep.

A study finds that noise exposure adversely affects sleep which could contribute if an individual lived near an airport, busy freeway or heavy industry. [28] In terms of ENSO, a Korean study found that precipitation reduced airborne PM_10_ particles, which suggests that ENSO-related PM_10_ changes in the Korean Peninsula might be associated with precipitation variability. [29] Depending on the region of the United States, an El Niño or La Niña year may increase storm activity and winds that result in transport of PM_2.5_ across large distances. Wind carried PM_2.5_ across long periods results the population to breathe polluted air which can get transported into the bloodstream and the brain, which may lead to neural inflammation, disturbing of the sleep cycle. [11].

There are many limitations pertaining to our study. First, our exposure and outcome data is annual time scales, yet air pollution [30], sleep issues [31], and ENSO [22] are known to vary across different seasons of the year. The timing of the correct season can increase the risk of extreme PM_2.5_ levels on insufficient sleep, hence using annual means may obscure acute effects from seasonal events. Secondly, because the data source of insufficient sleep acquires information through surveys, we may not be capturing the full county-level distribution in prevalence. Thirdly, while our outcome was age-standardized, older adults are more likely to experience insufficient sleep, possibly increasing the risk with age. Sampling bias may have occurred because vulnerable populations without phone access (e.g., institutionalized individuals, very low-income groups) may be underrepresented. There may be county-level spatial autocorrelation present in the data, which was not assessed as it was not the scope of this research question. We were also unable, due to data limitations, to incorporate other population level risk factors, such as race or ethnicity, socioeconomic status, poverty, and access to healthcare or health sleep facilities. [32] Future research should consider multi-pollutant (i.e. ozone, nitrous, and/or sulfur oxide) models to provide a more comprehensive understanding of the environmental factors affecting sleep patterns.

## Conclusion

The relationship between environmental and climate factors and insufficient sleep remains understudied even as rates of insufficient sleep are increasing in the United States and globally. Based on our cross-sectional analysis, long-term (i.e. annual) effects of air pollution can pose a risk on other sleep outcomes. ENSO was found to be a significant effect modifier. As an emerging body of research points to links between environmental and climate factors and health outcomes, further investigations are needed to understand the environmental effects on insufficient sleep, as climate change results in heightened climate variability that will directly and indirectly impact health outcomes at a population level.

## Data Availability

All data generated or analysed during this study are included in this published article.
